# Revisiting the MotionWatch8©: Calibrating Cut-Points for Measuring Physical Activity and Sedentary Behavior Among Adults With Stroke

**DOI:** 10.3389/fnagi.2019.00203

**Published:** 2019-08-05

**Authors:** Ryan S. Falck, John R. Best, Michael C. R. Li, Janice J. Eng, Teresa Liu-Ambrose

**Affiliations:** ^1^Department of Physical Therapy, The University of British Columbia, Vancouver, BC, Canada; ^2^Djavad Mowafaghian Centre for Brain Health, The University of British Columbia, Vancouver, BC, Canada; ^3^Rehabilitation Research Program, GF Strong Rehabilitation Centre, Vancouver, BC, Canada; ^4^Brain Research Centre, The University of British Columbia, Vancouver, BC, Canada

**Keywords:** accelerometers, actigraphy, indirect calorimeter, validation, sedentary behavior, physical activity, stroke

## Abstract

Reduced moderate-to-vigorous physical activity (MVPA) and increased sedentary behavior (SB) are common following stroke, which can limit stroke recovery and contribute to greater cognitive decline. Hence, the MVPA and SB of adults with stroke should be measured concurrently using objective methods. One currently available method for objectively measuring MVPA and SB is the MotionWatch8© (MW8). However, adults with stroke can have significant mobility restrictions (depending on stroke severity) and thus it is important to determine separate MVPA and SB cut-points for adults with stroke, as well as validate separate cut-points: (1) when the MW8 is worn on the stroke affected side compared to the non-affected side; and (2) for adults with mild stroke versus adults with moderate-to-severe stroke. In the current study, we concurrently measured MW8 actigraphy (worn on both the stroke affected side and the non-affected side) and indirect calorimetry during 10 different activities of daily living for 43 adults with stroke (aged 55–87 years). Using intra-class correlations (ICC), we first investigated the agreement of the MW8 when placed on the affected side as compared to the non-affected side for: (1) all participants irrespective of stroke severity; (2) participants with mild stroke, classified as a Fugl Meyer motor score of ≥79/100; and (3) participants with moderate-to-severe stroke (i.e., Fugl Meyer < 79/100). We then determined cut-points for all participants—as well as separate cut-points based on stroke severity—on both the stroke affected side and non-affected side for SB and MVPA using receiver operating characteristic curves. The results of our analyses indicate that the agreement in MW8 output between the stroke affected and non-affected sides was moderate across all participants (ICC = 0.67), as well as for each sub-group (mild stroke: ICC = 0.64; moderate-to-severe stroke: ICC = 0.77). Additionally, the results of our cut-point analyses support using different cut-points for different levels of stroke severity and also for the stroke affected side. We determined the following cut-points: (1) for the affected side, adults with mild stroke have cut-points of SB ≤134 counts per minute (CPM) and MVPA ≥704 CPM, while adults with moderate-to-severe stroke have cut-points of SB ≤281 CPM and MVPA ≥468 CPM; and (2) the non-affected side, adults with mild stroke have cut-points of SB ≤162 CPM and MVPA ≥661 CPM, while adults with moderate-to-severe stroke have cut-points of SB ≤281 CPM and MVPA ≥738 CPM. Hence, these data provide a new measure for concurrently examining the dynamic relationships between MVPA and SB among adults with stroke.

## Introduction

One new case of stroke occurs every 40 s in the United States alone (Go et al., 2014). Less than 50% of people who suffer a stroke will return to independent living within a year (Leys et al., 2005), and even those who regain functional independence will often continue to manifest significant physical and cognitive deficits (Hochstenbach et al., 2005). Not only does stroke increase the risk of early death by twofold, but individuals with significant physical and cognitive impairments post-stroke have even greater mortality-risk and are more likely to suffer a second catastrophic stroke (Smajlovic et al., 2006). Thus, identifying modifiable risk-factors to maintain and possibly restore the health of adults post-stroke is critical.

Increasing physical activity (PA) may be critical to maintain and restore health post-stroke (McDonnell, 2010). Strong epidemiological evidence consistently indicates that engaging in ≥150 min/week of PA of at least 3.0 metabolic equivalents (METs) is associated with increased life span and reduced risk of chronic conditions including stroke, dementia, and diabetes mellitus (Blair and Brodney, 1999; Nelson et al., 2007; Blair, 2009; Hamer and Chida, 2009). While the importance of PA for health is well established, decreases in PA are a common consequence of stroke (Moore et al., 2013; English et al., 2014; Fini et al., 2017). For example, it is estimated that adults with stroke take over 4000 fewer steps per day than their healthy peers (Fini et al., 2017). Decreases in PA levels after stroke can also lead to disuse atrophy and cardiovascular deconditioning, which combined with social isolation and associated psychological factors can lead to an increased risk for secondary cardiac complications and recurrent stroke (Billinger et al., 2014).

Reducing sedentary behavior (SB)—defined as any behavior that incurs ≤1.5 METs such as sitting, television watching, and lying down (Pate et al., 2008)—may also be an important modifiable risk factor following stroke. There is mounting evidence that high SB is linked to a greater risk of early death (Biswas et al., 2015), diabetes mellitus (Owen et al., 2010), cardiovascular disease (Ford and Caspersen, 2012), and cognitive decline (Falck et al., 2016a). Importantly, recent epidemiological data suggests adults with stroke spend more than 10 h/day in SB, and 1.2 h/day more than their non-stroke peers (Butler and Evenson, 2014). Although little is known about the consequences of high SB following a stroke, high SB is a risk factor for stroke independent of PA (Chomistek et al., 2013), which suggests that reducing SB among adults with stroke may help maintain the health of this population.

Importantly, there is increasing evidence that PA (and to a lesser extent SB) each play a key role in the preservation of cognitive health post-stroke. Considerable evidence indicates greater amounts of PA improves cognitive function and reduces dementia risk (Colcombe and Kramer, 2003; Hamer and Chida, 2009), and increasing PA among adults with stroke can significantly improve cognitive function (Cumming et al., 2012). Adults with higher SB also have poorer cognitive performance in later life (Falck et al., 2016a), and thus it is likely that high SB is a risk factor for further cognitive decline in adults with stroke. While PA and SB may each have important implications on cognitive health, there is mounting evidence that there is also a dynamic relationship between PA and SB (Katzmarzyk, 2010; Owen et al., 2010), which may directly influence cognitive function. It is thus important to concurrently examine the implications of each of these behaviors on cognitive health—and each other (Landry et al., 2015; Falck et al., 2016b).

Given the aforementioned evidence, and the knowledge that stroke doubles the risk of all-cause dementia (Savva and Stephan, 2010), the need to understand how PA and SB impacts the cognitive health of adults with stroke is clear. Doing so requires the availability of well-designed tools, with good evidence of validity and reliability, which are capable of measuring PA and SB concomitantly. One such measure is the MotionWatch8© (MW8), which has evidence of validity and reliability for measuring PA and SB in healthy older adults (Landry et al., 2015; Falck et al., 2016b). However, it is important that a measure have evidence of validity and reliability for the population which one intends to observe (Falck et al., 2015). Wrist-worn actigraphy has potential as a tool for measuring PA and SB in adults with stroke (Lee et al., 2018), however adults with stroke expend more energy during walking than their healthy peers (Kramer et al., 2016). It is thus plausible that not only does the MW8 require a different calibration for adults with stroke, but that the side of the body which the MW8 is worn, and the severity of stroke require different calibrations of the device. As a first step toward creating a valid and reliable measure for objectively monitoring PA and SB concomitantly in adults with stroke, we set out to (1) calibrate and validate cut-points for PA and SB for adults with stroke; (2) determine if different cut-points are required based on which side of the body the MW8 is placed; and (3) determine if different cut-points are required for adults with mild stroke as compared to adults with moderate-to-severe stroke.

## Materials and Methods

Ethical approval was obtained from the Vancouver Coastal Health Research Institute and the University of British Columbia’s Clinical Research Ethics Board (H13-00715). All participants provided written informed consent.

### Participants

Adults with chronic stroke (*N* = 43) were recruited from an ongoing 6-month proof-of-concept randomized controlled trial (RCT) to improve cognitive function in adults with stroke (Best et al., 2018). Participants were recruited from the RCT following baseline assessment. Briefly, our sample of adults with stroke consisted of community-dwelling men and women: (1) aged 55 years and older; (2) who had an ischemic or hemorrhagic stroke (confirmed by previous MRI or computed tomography scan) at least 1 year prior to study enrollment; (3) Mini-Mental State Examination (MMSE) score of ≥20/30 (Folstein et al., 1975); (4) able to read, write, and speak English with acceptable visual and auditory acuity; (5) not expected to start or are stable on a fixed dose of cognitive medications (e.g., donepezil, galantamine, etc.); (6) able to walk ≥6 m with rest intervals; (7) not currently participating in any regular therapy or progressive exercise; and (8) not diagnosed with dementia of any type, or another neurodegenerative or neurological condition. Individuals interested in participating in the study were pre-screened for eligibility criteria. We included individuals in the study who: (1) were cleared for exercise using the modified Physical Activity Readiness Questionnaire (Modified PAR-Q; Cardinal et al., 1996; Cardinal and Cardinal, 2000) and could walk independently with or without a walking aid. We excluded participants who were unable to wear a portable indirect calorimeter during testing.

### Measures

#### Demographics

Participants were surveyed via questionnaire for their age, sex, race, and ethnicity; as well as any current or previous health conditions such as diabetes, hypertension, osteoporosis, arthritis, and cancer. We recorded whether participants used a cane or other ambulatory device, and height and body weight (as measured by a calibrated stadiometer and electronic scale, respectively) were used to determine each participant’s body mass index (BMI; kg/m2). In addition, we assessed participant motor function using the Fugl Meyer motor score (Fugl-Meyer et al., 1975; Gladstone et al., 2002). Participants were scored on a scale of 0–100; 0–66 for the upper body, and 0–34 for the lower body.

#### Wrist Worn Accelerometer

We used the MW8 actigraphy system (cam*n*tech): a light weight, water-proof, tri-axial wrist-worn accelerometer. The protocol in our previous MW8 validation study was to place the device on the non-dominant wrist (a standard procedure; Landry et al., 2015), however, we modified this protocol such that participant wore a MW8 on each wrist (i.e., stroke affected side and non-affected side). MW8 data were recorded using 60 s epochs.

#### Indirect Calorimeter

As our criterion measure, we used the Cosmed k4b2, a portable indirect calorimeter (Cosmed; Rome, Italy) to determine energy expenditure during each assessment. Indirect calorimetry is used to measure oxygen uptake (VO_2_) and production of carbon dioxide (VCO_2_). Inspired and expired gases are collected via a breathing mask and then analyzed to determine VO_2_ and VCO_2_ volumes. These measurements are used to determine energy expenditure in the form of metabolic equivalents (METs) via Schofield-equations (Schofield, 1985). Briefly, Schofield-equations estimate metabolic rate according to weight, age and sex; these estimates of metabolic rate are then used to classify an activity based on the METs it requires. In accordance with established guidelines (Pate et al., 2008), activities were classified as follows: (1) sedentary (<1.5 METs); (2) light (1.5–3.0 METs), or moderate-to-vigorous PA (MVPA; >3.0 METs).

### Measurement Protocol

The Cosmed gas analyzers were calibrated and verified with known gases immediately before and after each test. Following calibration completion, the Cosmed was fitted for comfort to each participant. The Cosmed was worn concurrently with MW8’s equipped on the wrist of the stroke affected side and the non-affected side.

Measurement of METs was recorded at 60 s intervals on the indirect calorimeter. The METs data from each session were downloaded into Microsoft Excel for further processing. To ensure consistency across measurement sessions, two assessors (RSF and MCRL) – who were previously trained on all aspects of the measurement protocol – calibrated the Cosmed and conducted the trials for all participants. All instruments were synchronized to the same clock, and time was recorded at the beginning and end of each activity to ensure appropriate data comparisons could be made across recording devices. For all sessions, notes on participant activity during the trial were documented.

The measurement session lasted ∼60 min, during which each participant performed 10 different activities designed to mimic activities of daily living: (1) walking at four different paces; (2) sitting in a chair; (3) cleaning; (4) resistance training; (5) lying down; and (6) standing. To more closely mimic free-living activity, participants were allowed to move their arms freely during these activities, however, the measurement session occurred in a quiet room. Participants were provided standardized instructions (as per a predetermined script), prior to beginning each activity. As described below, participants performed 10 activities – for 5 min each – in the following order:

#### Walking

Participants were asked to walk at four different speeds around a circular hallway (∼50 meters in circumference), with each speed intended to mimic a specific pace: *Leisurely, Comfortable, Moderate*, and *Brisk*. Participants self-selected the speed for each pace, as described below. This was done for safety reasons, as well as to adjust for variability in fitness level across participants. Once the speed had been selected for each pace, the participant was asked to maintain this pace for 5 min. While we did not require participants to be able to walk 5 min without stopping, only two participants required a break of <1 min during the *Brisk Pace* condition. After walking for 5 min at a given pace, participants were given a 2 min recovery period, during which they could sit down. The following order was used for each self-selected pace:

##### Leisurely pace

Participants were first instructed to walk at a leisurely pace. This speed was described as a pace the participant would walk at during a casual walk with a friend. Participants were instructed this pace was supposed to be easy, requiring minimal exertion.

##### Comfortable pace

Participants were instructed to walk at a pace they would use for a little light exercise. This walking speed was described as being faster than the previous speed, but requiring limited exertion.

##### Moderate pace

Participants were instructed to walk at a pace they would use for moderate exercise or when completing an errand. Participants were instructed to walk at a quick pace, so they would walk with purpose but not urgency.

##### Brisk pace

This speed was described as a pace the participant would walk if they were running late or needed to get somewhere as fast as possible. It was intended to be the fastest pace the participant could walk at for 5 min without slowing down.

#### Sitting

Participants were instructed to sit in a chair. No instructions were given as to how they must sit, but they were asked to remain seated for the entire 5 min. Typically, the experimenter and participant engaged in polite conversation during this time.

#### Cleaning

Participants were led to a sink, where they were provided soap to wash three plates and three cups. Participants were told to wash the provided dishes as they normally would in their own home. Participants were asked to wash the provided dishes continuously for the duration of 5 min.

#### Resistance Training

Participants engaged in two resistance training activities that are typical for resistance training programs: Bicep Curls and Chair Squats. Participants completed three sets of 10 repetitions for each exercise. Rest between sets of each exercise was permitted as needed.

##### Bicep curls

Instructions were provided as needed to ensure participants performed all bicep curls using proper technique in a slow and controlled manner while sitting. Five-pound dumbbells and ten-pound dumbbells were used by women and men, respectively. In cases where a participant was unable to complete bicep curls with the stroke affected arm, the participant was asked (to the best of their ability) to grip one dumbbell with both hands and perform a bicep curl with both hands holding the dumbbell.

##### Chair squats

This required participants to sit down in a chair and then stand up to complete one repetition. Participants did not use weights for this task, and were allowed to use the arm rests of the chair as needed.

#### Lying Down

Participants were instructed to lie down on a bed for a period of 5 min.

#### Standing

Participants were asked to remain standing for 5 min, but were given the option to move around the room freely. No additional instructions were given during this time as this period was intended to mimic standing and free-movement as part of routine daily activity. Following completion of this activity, the trial was terminated.

### Statistical Analysis

We performed all statistical analyses in R version 3.3.2 using the *readxl, pastecs, psych, plyr, pROC*, and *BlandAltmanLeh* packages. Our statistical code is available in Supplementary Material S1.

#### Stratification and Demographics

In order to account for potential differences in motor function due to stroke severity, we stratified participants based on Fugl Meyer motor score according to the criteria of Duncan et al. (1994). We calculated means and standard deviations for all variables of interest for all participants, and then separately for participants with mild stroke (Fugl Meyer ≥ 79/100; *N* = 29) or moderate-to-severe stroke (Fugl Meyer < 79; *N* = 14). We then performed independent samples *t*-tests and chi-square tests to determine group differences in demographic variables of interest.

#### Agreement Analyses

We determined the level of agreement of the MW8 when placed on the stroke affected side as compared to the non-affected side. Data from the left and right MW8 were summarized and compared using partial Pearson correlations (*r*) wherein we controlled for the participant assessed, and intra-class correlations (ICC; [3, *k*]) to assess average agreement between the MW8 placed on the stroke affected side and the MW8 placed on the non-affected side over different levels of activity. A higher *r* and/or ICC is indicative of greater agreement. We categorized the level of agreement according to the criteria of Koo and Li (2016). In addition, we generated Bland-Altman plots to determine agreement over different intensities of activity between the MW8 placed on the stroke affected side and the MW8 placed on the non-affected side (Bland and Altman, 1986). We performed these agreement analyses for all participants, as well as stratified based on stroke severity (i.e., mild stroke vs. moderate-to-severe stroke).

#### Determining Optimal Cut-Points for the MW8

Our primary objective was to derive MW8 activity count cut-points for adults with stroke for SB, light PA (LPA), and moderate-to-vigorous PA (MVPA). We derived two separate sets of cut-points for the MW8—one for the stroke affected side, and one for the non-affected side. For each set of cut-points, receiver operator characteristics (ROC) analyses (Berk, 1976) were performed to determine optimal cut-points for the following intensities: SB (<1.5 METs), LPA (1.5 – 3.0 METs); and MVPA (>3.0 METs) (National Institutes of Health, 2008; Pate et al., 2008). For each ROC curve, METs were coded as 0 or 1 according to the cut-point being established. For example, when the sedentary cut-point was being established, a “1” was assigned to all minutes wherein a participant was reported in SB via indirect calorimetry and a “0” was assigned to all minutes wherein a participant was not reported in SB based on indirect calorimetry. The area under the ROC curve (AUC) was calculated for both SB and MVPA. We categorized the diagnostic accuracy of the AUC using the criteria of Fischer et al. (2003). In addition, we determined whether the diagnostic accuracy of the AUC was significantly different between the non-affected and the affected sides using DeLong’s test for two correlated ROC curves (DeLong et al., 1988).

*Sensitivity, Specificity, Accuracy, Positive Predictive Value*, and *Negative Predictive Value* were calculated for both the SB and MVPA cut-points. *Sensitivity* is the percentage of epochs correctly identified as being engaged at the activity level examined (e.g., percent of actual MVPA epochs correctly identified as an epoch of MVPA). *Specificity* is the percentage of epochs correctly identified as not being engaged at the activity level examined (e.g., percent of actual non-MVPA epochs correctly identified as not being an epoch of MVPA). *Accuracy* is defined as the percent of correct decisions made using the derived cut-point (e.g., percent correctly identified as MVPA and percent correctly identified as non-MVPA). *Positive Predictive Value* is the probability of correctly identifying an epoch as being engaged at that activity level, given that it truly is an epoch of that activity level (e.g., probability of correctly identifying an epoch of MVPA as MVPA). *Negative Predictive Value* is the probability of correctly identifying an epoch as not being engaged at that activity level, given that it truly is not an epoch of that activity level (e.g., the probability of correctly identifying an epoch of sedentary activity as not being MVPA).

Optimal cut-points were established for SB using the Youden index (*J*; Akobeng, 2007). Briefly, *J* is defined as the maximum vertical distance between the ROC curve and the diagonal (i.e., chance line) and is calculated as *J* = maximum [sensitivity + specificity – 100]. Using this measure, the maximum value for *J* is considered to be the optimal cut-point. Cut-points for MVPA were established using the highest *J* given a false-positive-ratio < 0.10. We derived cut-points using this approach to prevent overestimation and provide a more conservative estimate of MVPA. The cut-point for LPA was determined as the activity level between the boundaries for SB and MVPA.

After determining separate cut-points for each activity type for all participants, we then proceeded to develop separate cut-points based on stroke severity (i.e., mild stroke vs. moderate-to-severe stroke). Thus, we derived six separate sets of cut-points: two for all participants (i.e., stroke affected side or non-affected side), two for participants with mild stroke, and two for participants with moderate-to-severe stroke.

## Results

### Participant Characteristics

Participant characteristics are described in Table 1. Mean participant age was 67 years (*SD* = 7 years), mean BMI was 27.17 kg/m^2^ (*SD* = 5.25 kg/m^2^) and 35% of our sample was female. In addition, 56% of our sample required a cane or a walker. Average Fugl Meyer motor score for the sample was 78 (*SD* = 22; Range: 8–98). When we stratified participants according to stroke severity, there were no significant group differences in age, BMI, or sex. Participants with moderate-to-severe stroke had significantly poorer Fugl Meyer upper body motor score (Moderate-to-Severe Stroke: 30 [*SD* = 17]; Mild Stroke: 62 [*SD* = 5]; *p* < 0.01) and significantly poorer total score (Moderate-to-Severe Stroke: 54 [*SD* = 22]; Mild Stroke: 90 [*SD* = 5]; *p* < 0.01).

**TABLE 1:** Participant characteristics (*N* = 43).

Variable	All Participants (*N* = 43)	Adults with Moderate-to-Severe Stroke (*N* = 14)	Participants with Mild Stroke (*N* = 29)	*p-value*
Age	67 (7)	70 (9)	67 (6)	0.20
BMI (kg/m^2^)	27.17 (5.25)	26.63 (2.66)	27.43 (6.15)	0.55
% Female	35%	29%	38%	0.79
**Race**				
*Caucasian*	72%	64%	76%	0.60
*Asian*	14%	14%	14%	
*Other*	14%	22%	10%	
**Comorbidities**				
*Diabetes Mellitus*	19%	36%	10%	0.13
*Cancer*	14%	14%	14%	0.99
*Osteoporosis*	14%	14%	14%	0.99
**Mobility assistance**				
*Use a cane*	56%	64%	48%	0.20
*Use a walker*	2%	7%	0%	
**Fugl Meyer Motor Score**				
*Upper Body (66 points)*	51 (18)	30 (17)	62 (5)	<0.01
*Lower Body (34 points)*	27 (6)	24 (8)	29 (4)	0.07
*Total Score (100 points)*	78 (22)	54 (22)	90 (5)	<0.01

### Agreement Analyses

Table 2 describes the agreement of the MW8 when placed on the stroke affected side as compared to the non-affected side for all participants, adults with mild stroke, and adults with moderate-to-severe stroke.

**TABLE 2:** Agreement of the MotionWatch8 for adults with stroke.

	Watch Side	Number of Epochs	Mean counts per minute (*SD*) [Range]	Pearson Correlation	Interclass Correlations (95% CI)
All Participants (*N* = 43)	Stroke Affected Side	2277	377 (443) [0–3617]	0.53	0.68 (0.65, 0.70)
	Non-Affected Side	2277	408 (396) [0–2591]	–	–
Adults with Mild Stroke (*N* = 29)	Stroke Affected Side	1527	410 (469) [0–3617]	0.51	0.65 (0.61, 0.68)
	Non-Affected Side	1527	392 (381) [0–2591]	–	–
Adults with Moderate-to-Severe Stroke (*N* = 14)	Stroke Affected Side	750	310 (374) [0–2043]	0.60	0.77 (0.73, 0.80)
	Non-Affected Side	750	440 (421) [0–2318]	–	–

#### All Participants (*N* = 43*)*

**FIGURE 1:**
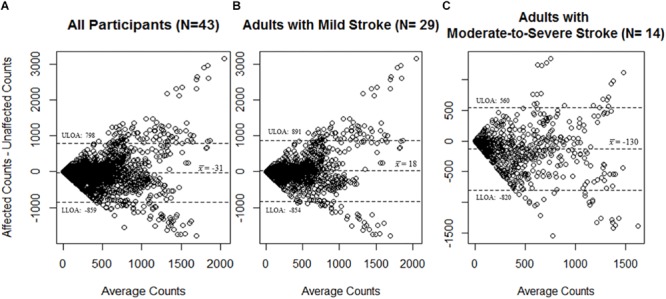
Bland–Altman plots for agreement of the MotionWatch8 for **(A)** all participants (*N* = 43); **(B)** participants with mild stroke (*N* = 29); and **(C)** participants with moderate-to-severe stroke (*N* = 14). Differences between counts reported by the stroke affected side MW8 and the stroke non-affected side MW8 are reported. A greater amount of activity was associated with less agreement between the affected side MW8 and non-affected side MW8, irrespective of stroke severity. Dotted lines represent upper and lower limits of agreement. ULOA: Upper Limit of Agreement; LLOA: Lower Limit of Agreement.

A total of 2277 total epochs were captured for all participants on each of the MW8s worn (i.e., on the wrist of the stroke affected side and on the wrist of the non-affected side). There was a moderate correlation between CPM measured by each of the MW8 (*r* = 0.53). The ICC assessing consistency of the measures provided by each MW8 indicated moderate agreement (ICC = 0.68; 95% CI [0.65, 0.70]). As shown in Figure 1, the average difference between MW8s (i.e., stroke affected side – non-affected side) was -31 CPM (*SD* = 414 CPM). In addition, there was greater disagreement between the affected side and the non-affected side with higher CPM.

#### Adults With Mild Stroke (*N* = 29)

Among participants with mild stroke, we collected 1527 epochs on each of the MW8s worn (Table 2). There was a moderate correlation between CPM measured by each of the MW8 (*r* = 0.51). The ICC assessing consistency of the measures provided by each MW8 indicated moderate agreement (ICC = 0.65; 95% CI [0.61, 0.68]). The average difference between MW8s was 18 CPM (*SD* = 436 CPM), as detailed in Figure 1, and there was greater disagreement between the affected side and the non-affected side with higher CPM.

#### Adults With Moderate-to-Severe Stroke (*N* = 14)

Among participants with moderate-to-severe stroke, we collected 750 epochs on each of the MW8s worn (Table 2). There was a moderate correlation between CPM measured by each of the MW8 (*r* = 0.60). The ICC assessing consistency of the measures provided by each MW8 indicated good agreement (ICC = 0.77; 95% CI [0.73, 0.80]). The average difference between MW8s was −130 CPM (*SD* = 345 CPM; Figure 1). In addition, we found greater disagreement between the affected side and the non-affected side with higher CPM.

### ROC Curve Analyses

#### ROC Curve Analysis for All Participants

**FIGURE 2:**
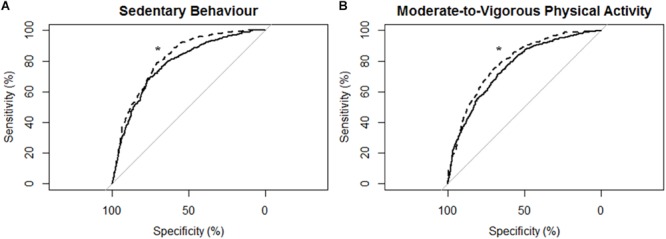
Receiver operating characteristic (ROC) curves and corresponding area under the curves (with 95% confidence interval; AUC) for all participants (*N* = 43) with the MotionWatch8 (MW8) placed on either the non-affected side or the stroke affected side. **(A)** ROC curve for sedentary behavior with MW8 placed on the non-affected side (–) vs. affected side (- - -). We found there was moderate diagnostic accuracy for the non-affected side (AUC = 0.777; 95% CI: [0.754–0.801]), and moderate diagnostic accuracy for the affected side (AUC = 0.814; 95% CI: [0.794–0.834]). **(B)** ROC curve for moderate-to-vigorous physical activity with MW8 placed on the non-affected side (–) vs. affected side (- - -). We found there was moderate diagnostic accuracy for the non-affected side (AUC = 0.758; 95% CI: [0.738 – 0.779]), and moderate diagnostic accuracy for the affected side (AUC = 0.792; 95% CI: [0.773–0.810]). ^*^Significant difference in diagnostic accuracy between ROC curves (DeLong et al., 1988).

Figure 2 describes the ROC curves for SB and MVPA respectively for all participants. On the non-affected side, the AUC for the ROC curve was moderately accurate for SB (AUC = 0.78; 95% CI [0.75, 0.80]) and moderately accurate for MVPA (AUC = 0.76; 95% CI [0.74, 0.78]). On the stroke affected side, the AUC for the ROC curve was moderately accurate for SB (AUC = 0.81; 95% CI [0.79, 0.83]) and moderately accurate for MVPA (AUC = 0.79; 95% CI [0.77, 0.81]). The AUC for the stroke affected side was significantly greater for both the ROC curve for SB (*Z* = 3.47; *p* < 0.01) and MVPA (*Z* = 3.83; *p* < 0.01).

#### ROC Curve Analysis for Adults With Mild Stroke

**FIGURE 3:**
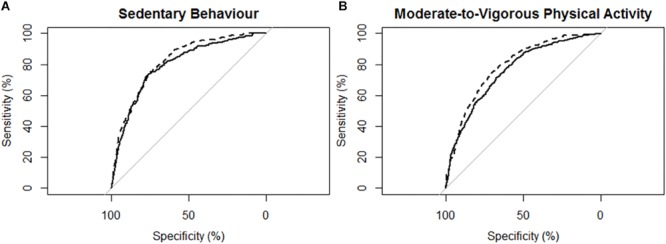
Receiver operating characteristic (ROC) curves and corresponding area under the curves (with 95% confidence interval; AUC) for participants with mild stroke (*N* = 29) with the MotionWatch8 (MW8) placed on either the non-affected side or the stroke affected side. **(A)** ROC curve for sedentary behavior with MW8 placed on the non-affected side (–) vs. affected side (- - -). We found there was moderate diagnostic accuracy for the non-affected side (AUC = 0.795; 95% CI: [0.766 – 0.823]), and moderate diagnostic accuracy for the affected side (AUC = 0.820; 95% CI: [0.795–0.845]). **(B)** ROC curve for moderate-to-vigorous physical activity with MW8 placed on the non-affected side (–) vs. affected side (- - -). We found there was moderate diagnostic accuracy for the non-affected side (AUC = 0.759; 95% CI: [0.735–0.784]), and moderate diagnostic accuracy for the affected side (AUC = 0.766; 95% CI: [0.742–0.790]).

Figure 3 describes the ROC curves for SB and MVPA respectively for participants with mild stroke. The AUC on the non-affected side for the ROC curve was moderately accurate for SB (AUC = 0.80; 95% CI [0.77, 0.82]) and moderately accurate for MVPA (AUC = 0.76; 95% CI [0.74, 0.78]). The AUC on the stroke affected side was moderately accurate for SB (AUC = 0.82; 95% CI [0.80, 0.85]) and moderately accurate for MVPA (AUC = 0.77; 95% CI [0.74, 0.79]). The AUC for the stroke affected side was marginally greater for the ROC curve of SB (Z = 1.959; *p* = 0.051), but was not significantly different for the ROC curve of MVPA (Z = 0.69; *p* = 0.49).

#### ROC Curve Analysis for Adults With Moderate-to-Severe Stroke

**FIGURE 4:**
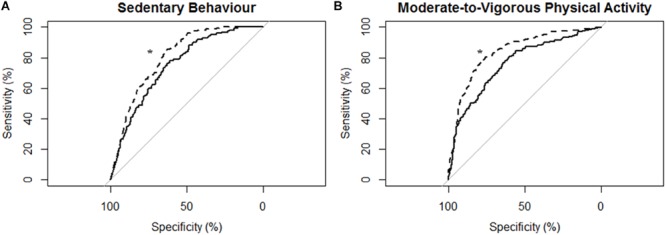
Receiver operating characteristic (ROC) curves and corresponding area under the curves (with 95% confidence interval; AUC) for participants with moderate-to-severe stroke (*N* = 14) with the MotionWatch8 (MW8) placed on either the non-affected side or the stroke affected side. **(A)** ROC curve for sedentary behavior with MW8 placed on the non-affected side (–) vs. affected side (- - -). We found there was moderate diagnostic accuracy for the non-affected side (AUC = 0.748; 95% CI: [0.708–0.788]), and moderate diagnostic accuracy for the affected side (AUC = 0.804; 95% CI: [0.771–0.837]). **(B)** ROC curve for moderate-to-vigorous physical activity with MW8 placed on the non-affected side (–) vs. affected side (- - -). We found there was moderate diagnostic accuracy for the non-affected side (AUC = 0.760; 95% CI: [0.723–0.797]), and moderate diagnostic accuracy for the affected side (AUC = 0.839; 95% CI: [0.809–0.869]). ^*^Significant difference in diagnostic accuracy between ROC curves (DeLong et al., 1988).

Figure 4 describes the ROC curves for SB and MVPA respectively for participants with moderate-to-severe stroke. The AUC on the non-affected side was moderately accurate for SB (AUC = 0.75; 95% CI [0.71, 0.79]) and moderately accurate for MVPA (AUC = 0.76; 95% CI [0.72, 0.80]). The AUC on the stroke affected side was moderately accurate for SB (AUC = 0.80; 95% CI [0.77, 0.84]) and moderately accurate for MVPA (AUC = 0.84; 95% CI [0.81, 0.87]). The AUC for the stroke affected side was significantly greater for both the ROC curve for SB (Z = 3.16; *p* < 0.01) and MVPA (Z = 4.96; *p* < 0.01).

### Determining Optimal MW8 Cut-Points for Adults With Stroke

Table 3 describes the optimum cut-points for SB and MVPA with LPA being between those boundaries.

**TABLE 3:** Cut-points for MotionWatch8 dependent on wrist-placement.

	Activity Level	Cut-point	Sensitivity	Specificity	Accuracy	Youden Index	Positive Predictive Value	Negative Predictive Value
All Participants (*N* = 43)	**Non-affected Side**							
	*Sedentary*	≤165	69%	75%	74%	44.33	40%	91%
	*Light*	165 < *x* < 685	–	–	–	–	–	–
	*MVPA*	≥685	38%	91%	73%	27.31	69%	74%
	**Affected side**							
	*Sedentary*	≤134	79%	71%	72%	49.35	39%	93%
	*Light*	134 < *x* < 652	–	–	–	–	–	–
	*MVPA*	≥652	41%	90%	73%	30.76	68%	74%
Adults with Mild Stroke (*N* = 29)	**Non-affected Side**							
	*Sedentary*	≤162	73%	76%	76%	49.53	42%	92%
	*Light*	162 < *x* < 661	–	–	–	–	–	–
	*MVPA*	≥ 661	34%	90%	71%	24.70	66%	72%
	**Affected Side**							
	*Sedentary*	≤134	75%	75%	75%	50.33	41%	93%
	*Light*	134 < *x* < 704	–	–	–	–	–	–
	*MVPA*	≥704	34%	90%	71%	24.13	65%	72%
Adults Moderate-to-Severe Stroke (*N* = 14)	**Non-affected Side**							
	*Sedentary*	≤281	78%	61%	64%	39.00	33%	92%
	*Light*	281 < *x* < 738	–	–	–	–	–	–
	*MVPA*	≥738	43%	90%	74%	32.57	68%	76%
	**Affected Side**							
	*Sedentary*	≤123	85%	63%	67%	48.33	37%	95%
	*Light*	123 < *x* < 468	–	–	–	–	–	–
	*MVPA*	≥468	56%	90%	79%	46.13	74%	81%

#### Cut-Points for All Participants

For all participants independent of stroke severity, the cut-point for SB determined using the highest *J* was ≤165 CPM (*J* = 44.33) for the non-affected side and ≤134 CPM (*J* = 49.35) for the stroke affected side. After adjusting to target a false-positive ratio of ≤0.10, the derived cut-point for MVPA for the non-affected and stroke affected sides were ≥685 CPM (*J* = 27.31) and ≥652 CPM (*J* = 30.76), respectively.

#### Cut-Points for Adults With Mild Stroke

For adults with mild stroke, the SB cut-point determined using the highest *J* was ≤ 162 CPM (*J* = 49.53) for the non-affected side and ≤134 CPM (*J* = 50.33) for the stroke affected side. After adjusting to target a false-positive ratio of ≤0.10, the cut-point for MVPA for the non-affected side was ≥661 CPM (*J* = 24.70) and the cut-point for the stroke affected side was ≥704 CPM (*J* = 24.13).

#### Cut-Points for Adults With Moderate-to-Severe Stroke

The cut-points for SB for adults with moderate-to-severe stroke were ≤281 CPM (*J* = 39.00) and ≤123 CPM (*J* = 48.33), for the non-affected side and the stroke affected side respectively. After adjusting to target a false-positive ratio of ≤0.10, the cut-point for MVPA for the non-affected side was ≥738 CPM (*J* = 32.57) and the cut-point for the stroke affected side was ≥468 CPM (*J* = 46.13).

## Discussion

The present study provides the first evidence of agreement and validity for estimating PA and SB using the MW8 among adults with stroke. Our results indicate that separate cut-points are needed dependent on whether the MW8 is placed on the stroke affected side or the non-affected side, and that adults with mild motor impairment from stroke should require different cut-points than adults with greater impairment.

While there is some evidence that wrist-worn accelerometers produces less accurate estimates of PA and SB than hip-worn accelerometers (Troiano et al., 2014), the MW8 presents several distinct advantages as a measure for observing PA and SB (Landry et al., 2015; Falck et al., 2016b). The MW8 is waterproof and does not need to be removed during activities such as bathing or swimming. The MW8 is specifically designed for comfortable, continuous wear during the day and at night, which improves wearer compliance and minimizes missing data. For example, in a cross-sectional study of 152 older adults (Falck et al., 2017), our laboratory only determined two instances of non-wear time with the MW8 according to the criteria of Hutto et al. (2013). Finally, the MW8 might capture light activities better (i.e., household chores), especially those that do not involve the lower extremities.

Standard practice for measuring PA and SB using wrist-worn accelerometers has been to place the device on the non-dominant side (Crouter et al., 2014; Johansson et al., 2015). However, this protocol is challenging for adults with stroke if they have significant motor impairments since they may not be able to remove the device (if needed) or need to press an event-marker time stamp, which is often used to determine wake time from sleep time. Previous investigations of adults with stroke have thus used wrist-worn accelerometers on both the affected and non-affected side (Schuiling et al., 2005; Bakken et al., 2011; Noorkõiv et al., 2014); however, these studies were not interested in PA, but rather sleep quality and upper limb movement. Recent work does indicate that wrist-worn accelerometers worn on either the stroke affected or non-affected side may be useful for examining PA of adults with acute stroke in an in-patient setting (Lee et al., 2018), but our study is the first to our knowledge which has examined the agreement and validity of wrist-worn accelerometers for measuring PA and SB among adults with chronic stroke.

While it is thus pragmatic to place the MW8 on the side of the body which improves wearability, the results of our agreement analyses indicate that the MW8 provides significantly different estimates of activity based on the side of the body on which the device is placed. Specifically, our Bland–Altman plots (Figure 1) indicate that the device (1) has less consistency between the affected and non-affected sides as the activity intensity increases; and (2) has less consistency between the affected and non-affected sides for adults with moderate-to-severe stroke, as compared to adults with mild stroke. Given that the MW8 only has moderate agreement between the affected and non-affected sides according to the criteria of Koo and Li (2016), researchers should try (whenever possible) to use a standardized protocol for determining which side of the body to place the MW8. This protocol should be based in part on the expected number of participants with significant impairments in mobility from stroke. Moreover, it is important that researchers determine the stroke severity of participants at baseline using the Fugl-Meyer (Fugl-Meyer et al., 1975; Gladstone et al., 2002).

The results of our cut-point analyses also suggest that researchers should carefully consider whether to place the MW8 on the affected or non-affected side of the body. While using the non-affected vs. affected side does not appear to significantly impact diagnostic accuracy of SB and MVPA for adults with mild stroke, using our cut-points for the affected side in adults with moderate-to-severe stroke appears to significantly improve diagnostic accuracy of SB and MVPA. Moreover, should researchers not determine stroke severity *a priori*, it appears that using the stroke affected side to estimate SB and MVPA will provide more accurate estimates of these behaviors.

Interestingly, our ICC results indicate that the MW8 has more consistent readings between the affected and non-affected sides in adults with moderate-to-severe stroke. One plausible explanation for this finding, as illustrated in Table 2, is the ranges of CPM (on both the affected and non-affected sides) for adults with moderate-to-severe stroke is considerably less than the range of CPM for adults with mild stroke. Given the similar ranges of CPM for the affected and non-affected sides in adults with moderate-to-severe stroke, it is likely that the agreement between the stroke affected and non-affected sides would be considerably more than for adults with mild stroke since the range of possible values is much smaller. However, our Bland–Altman plots (Figure 1) illustrate that on average the affected side registers fewer CPM than the non-affected side for adults with moderate-to-severe stroke; there does not appear to be large differences in CPM between the affected and non-affected sides for adults with mild stroke. It is thus possible that while the consistency between the non-affected side and affected side is better for adults with moderate-to-severe stroke, there are considerable differences between the CPM output of the non-affected side and the affected side, and thus the MW8 requires different cut-points for both sides.

Our study thus provides an important step forward in the measurement of PA and SB for adults with stroke, since we provide validated cut-points for each of these behaviors using the MW8. Based on our findings, future investigations should consider both the side of the body on which the device is placed, as well as the severity of stroke when determining which of our cut-points to use.

### Limitations

Our study does have several important limitations. Our cut-points may be limited by inter-individual differences in physiological stress between participants. Numerous types of stressors including chronic illnesses, such as stroke, can affect energy expenditure (for review, see Preiser et al., 2015). The MET definitions of SB and MVPA may differ for the population of adults with stroke, since adults with stroke typically have increased energy costs during walking and other activities of daily living (Kramer et al., 2016). While wrist-worn accelerometry is a promising strategy for estimating PA and SB in adults with stroke since it may help improve wearability and compliance, use of these devices is not without limitations—particularly for measuring activity of the lower extremities (for review, see Gebruers et al., 2010; Noorkõiv et al., 2014). For example, wrist-worn accelerometers often measure unintentional activity which may be incorrectly classified as PA.

As we have previously acknowledged (Landry et al., 2015), the MW8 cut-points for PA and SB in healthy older adults lack the accuracy previously reported for the GENEA accelerometer (Esliger et al., 2011); this is also likely true for adults with stroke. In addition, it is unclear how MW8 CPM are calibrated (e.g., do they reflect total motion CPM on a treadmill), and thus future research should determine whether CPM reflect (1) trunk movement while walking; (2) trunk movement with arm swing; or (3) arm swing but no trunk movement, and whether stroke-related paralysis effects CPM output. We did not account for whether VO_2_ had reached steady state in our analyses, which limit our ability to accurately predict whether an individual is actually engaged in SB or PA. It is also unclear how well MW8 CPM relates to energy expenditure, and whether the device more accurately predicts energy expenditure with the non-affected side as compared to the affected side in adults with stroke. Future work should thus examine the accuracy of the MW8 during different activities and energy expenditures.

Our primary aim was to establish cut-points for estimating SB and MVPA in adults with stroke using the MW8. While our secondary analyses examined whether different cut-points are needed based on stroke severity and which side of the body the device is placed, these analyses were exploratory in nature. We therefore did not perform an *a priori* sample size calculation, and thus cannot determine whether our secondary analyses were adequately powered to detect differences in diagnostic accuracy. We also did not examine the test-retest reliability of the MW8, and thus future studies should examine the repeatability of the estimates of SB and MVPA.

Standard practice is to ask participants to fast prior to indirect calorimetry assessment (Bouten et al., 1994; Nichols et al., 2000; Schmitz et al., 2005), however, because of concern for participant safety we did not ask participants to fast prior to the measurement session. Hence, it is possible that the accuracy of the MW8 for adults with stroke is less than that of other available devices, although the MW8’s utility as a valid objective measure of both PA and SB, still make it an attractive tool for researchers working with adults with stroke. We expect the MW8 to be used frequently in future studies, and thus our cut-points should provide for increased depth of analysis in these studies.

Generalizability of our findings is also limited to MW8 use in adults with stroke. Nonetheless, we examined a heterogeneous sample of adults with stroke, both in terms of stroke type and location as well as motor impairment. Lastly, while we included adults with severe stroke as defined by the criteria of Duncan et al. (1994), our sample was of community-dwelling adults with stroke. Adults with stroke who have even more severe symptoms of stroke (e.g., tremor, severe paralysis, dysmetria, etc.) may require other measurement procedures than actigraphy to accurately estimate PA and SB.

## Ethics Statement

This study was carried out in accordance with the recommendations of the Vancouver Coastal Health Research Institute and the University of British Columbia’s Clinical Research Ethics Board (H13-00715), with written informed consent from all subjects. All subjects gave written informed consent in accordance with the Declaration of Helsinki. The protocol was approved by the University of British Columbia’s Clinical Research Ethics Board.

## Author Contributions

RF wrote the first draft of the manuscript. ML and RF conducted all the data collections and analyzed the data. RF and TL-A conceived the study concept and design. All authors wrote the portions of the manuscript and provided the critical review.

## Conflict of Interest Statement

The authors declare that the research was conducted in the absence of any commercial or financial relationships that could be construed as a potential conflict of interest.

## References

[B1] Akobeng A. K. (2007). Understanding diagnostic tests 3: receiver operating characteristic curves. *Acta Paediatr.* 96 644–647. 10.1111/j.1651-227.2006.00178.x 17376185

[B2] Bakken L. N. Lee K. A. Kim H. S. Finset A. Lerdal A. (2011). Sleep-wake patterns during the acute phase after first-ever stroke. *Stroke Res. Treat.* 2011:936298. 10.4061/2011/936298 21776369 PMC3138134

[B3] Berk R. A. (1976). Determination of optional cutting scores in criterion-referenced measurement. *J. Exp. Educ.* 45 4–9. 10.1080/00220973.1976.11011567

[B4] Best J. R. Eng J. J. Davis J. C. Hsiung R. Hall P. A. Middleton L. E. (2018). Study protocol for Vitality: a proof-of-concept randomised controlled trial of exercise training or complex mental and social activities to promote cognition in adults with chronic stroke. *BMJ Open* 8:e021490. 10.1136/bmjopen-2018-021490 29550783 PMC5875626

[B5] Billinger S. A. Arena R. Bernhardt J. Eng J. J. Franklin B. A. Johnson C. M. (2014). Physical activity and exercise recommendations for stroke survivors: a statement for healthcare professionals from the American Heart Association/American stroke association. *Stroke* 45 2532–2553. 10.1161/STR.0000000000000022 24846875

[B6] Biswas A. Oh P. I. Faulkner G. E. Bajaj R. R. Silver M. A. Mitchell M. S. (2015). Sedentary time and its association with risk for disease incidence, mortality, and hospitalization in adults: a systematic review and meta-analysis. *Ann. Intern. Med.* 162 123–132. 10.7326/M14-1651 25599350

[B7] Blair S. N. (2009). Physical inactivity: the biggest public health problem of the 21st century. *Br. J. Sports Med.* 43 1–2. 19136507

[B8] Blair S. N. Brodney S. (1999). Effects of physical inactivity and obesity on morbidity and mortality: current evidence and research issues. *Med. Sci. Sports Exerc.* 31 (Suppl.) S646–S662. 10593541 10.1097/00005768-199911001-00025

[B9] Bland J. M. Altman D. G. (1986). Statistical methods for assessing agreement between two methods of clinical measurement. *Lancet* 1 307–310. 10.1016/s0140-6736(86)90837-8 2868172

[B10] Bouten C. V. Westerterp K. R. Verduin M. Janssen J. D. (1994). Assessment of energy expenditure for physical activity using a triaxial accelerometer. *Med. Sci. Sports Exerc.* 26 1516–1523. 7869887

[B11] Butler E. N. Evenson K. R. (2014). Prevalence of physical activity and sedentary behavior among stroke survivors in the United States. *Top. Stroke Rehabil.* 21 246–255. 10.1310/tsr2103-246 24985392 PMC4146341

[B12] Cardinal B. J. Cardinal M. K. (2000). Preparticipation physical activity screening within a racially diverse, older adult sample: comparison of the original and revised physical activity readiness questionnaires. *Res. Q. Exerc. Sport* 71 302–307. 10.1080/02701367.2000.10608910 10999267

[B13] Cardinal B. J. Esters J. Cardinal M. K. (1996). Evaluation of the revised physical activity readiness questionnaire in older adults. *Med. Sci. Sports Exerc.* 28 468–472. 10.1097/00005768-199604000-00011 8778552

[B14] Chomistek A. K. Manson J. E. Stefanick M. L. Lu B. Sands-Lincoln M. Going S. B. (2013). Relationship of sedentary behavior and physical activity to incident cardiovascular disease: results from the women’s health initiative. *J. Am. Coll. Cardiol.* 61 2346–2354. 10.1016/j.jacc.2013.03.031 23583242 PMC3676694

[B15] Colcombe S. Kramer A. F. (2003). Fitness effects on the cognitive function of older adults: a meta-analytic study. *Psychol. Sci.* 14 125–130. 10.1111/1467-9280.t01-1-01430 12661673

[B16] Crouter S. E. Flynn J. I. Bassett D. R. Jr. (2014). Estimating Physical activity in youth using a wrist accelerometer. *Med. Sci. Sports Exerc.* 47 944–951. 10.1249/mss.0000000000000502 25207928 PMC4362848

[B17] Cumming T. B. Tyedin K. Churilov L. Morris M. E. Bernhardt J. (2012). The effect of physical activity on cognitive function after stroke: a systematic review. *In. Psychogeriatr.* 24 557–567. 10.1017/s1041610211001980 21996131

[B18] DeLong E. R. DeLong D. M. Clarke-Pearson D. L. (1988). Comparing the areas under two or more correlated receiver operating characteristic curves: a nonparametric approach. *Biometrics* 44 837–845. 3203132

[B19] Duncan P. W. Goldstein L. B. Horner R. D. Landsman P. B. Samsa G. P. Matchar D. B. (1994). Similar motor recovery of upper and lower extremities after stroke. *Stroke* 25 1181–1188. 10.1161/01.str.25.6.1181 8202977

[B20] English C. Manns P. J. Tucak C. Bernhardt J. (2014). Physical activity and sedentary behaviors in people with stroke living in the community: a systematic review. *Phys. Therapy.* 94 185–196. 10.2522/ptj.20130175 24029302

[B21] Esliger D. W. Rowlands A. V. Hurst T. L. Catt M. Murray P. Eston R. G. (2011). Validation of the GENEA accelerometer. *Med. Sci. Sports Exerc.* 43 1085–1093. 10.1249/MSS.0b013e31820513be 21088628

[B22] Falck R. S. Davis J. C. Liu-Ambrose T. (2016a). What is the association between sedentary behaviour and cognitive function? A systematic review. *Br. J. Sports Med.* 5 800–811. 10.1136/bjsports-2015-095551 27153869

[B23] Falck R. S. Landry G. J. Brazendale K. Liu-Ambrose T. (2016b). Measuring physical activity in older adults using motionwatch 8© actigraphy: how many days are needed? *J. Aging Phys. Act* 25 51–57. 10.1123/japa.2015-0256 27281368

[B24] Falck R. S. Landry G. J. Best J. R. Davis J. C. Chiu B. K. Liu-Ambrose T. (2017). Cross-Sectional relationships of physical activity and sedentary behavior with cognitive function in older adults with probable mild cognitive impairment. *Am. J. Phys. Ther.* 97 975–984. 10.1093/ptj/pzx074 29029554 PMC5803762

[B25] Falck R. S. McDonald S. M. Beets M. W. Brazendale K. Liu-Ambrose T. (2015). Measurement of physical activity in older adult interventions: a systematic review. *Br. J. Sports Med.* 50 464–470. 10.1136/bjsports-2014-094413 26276362

[B26] Fini N. A. Holland A. E. Keating J. Simek J. Bernhardt J. (2017). How physically active are people following stroke? Systematic review and quantitative synthesis. *Phys. Therapy.* 97 707–717. 10.1093/ptj/pzx038 28444348

[B27] Fischer J. E. Bachmann L. M. Jaeschke R. (2003). A readers’ guide to the interpretation of diagnostic test properties: clinical example of sepsis. *Intensive Care Med.* 29 1043–1051. 10.1007/s00134-003-1761-8 12734652

[B28] Folstein M. F. Folstein S. E. McHugh P. R. (1975). “Mini-mental state”. A practical method for grading the cognitive state of patients for the clinician. *J. Psychiatr. Res.* 12 189–198. 1202204 10.1016/0022-3956(75)90026-6

[B29] Ford E. S. Caspersen C. J. (2012). Sedentary behaviour and cardiovascular disease: a review of prospective studies. *Int. J. Epidemiol.* 41 1338–1353. 10.1093/ije/dys078 22634869 PMC4582407

[B30] Fugl-Meyer A. R. Jääskö L. Leyman I. Olsson S. Steglind S. (1975). The post-stroke hemiplegic patient. 1. a method for evaluation of physical performance. *Scan. J. Rehabil. Med.* 7 13–31. 1135616

[B31] Gebruers N. Vanroy C. Truijen S. Engelborghs S. De Deyn P. P. (2010). Monitoring of physical activity after stroke: a systematic review of accelerometry-based measures. *Arch. phy. Med. Rehabil.* 91 288–297. 10.1016/j.apmr.2009.10.025 20159136

[B32] Gladstone D. J. Danells C. J. Black S. E. (2002). The fugl-meyer assessment of motor recovery after stroke: a critical review of its measurement properties. *Neurorehabil. Neural Repair* 16 232–240. 10.1177/154596802401105171 12234086

[B33] Go A. S. Mozaffarian D. Roger V. L. Benjamin E. J. Berry J. D. Blaha M. J. (2014). Heart disease and stroke statistics—2014 update: a report from the American Heart Association. *Circulation* 129:e28–e292. 24352519 10.1161/01.cir.0000441139.02102.80 PMC5408159

[B34] Hamer M. Chida Y. (2009). Physical activity and risk of neurodegenerative disease: a systematic review of prospective evidence. *Psychol. Med.* 39 3–11. 10.1017/S0033291708003681 18570697

[B35] Hochstenbach J. Prigatano G. Mulder T. (2005). Patients’ and relatives’ reports of disturbances 9 months after stroke: subjective changes in physical functioning, cognition, emotion, and behavior. *Arch. Phys. Med. Rehabil.* 86 1587–1593. 10.1016/j.apmr.2004.11.050 16084812

[B36] Hutto B. Howard V. J. Blair S. N. Colabianchi N. Vena J. E. Rhodes D. (2013). Identifying accelerometer nonwear and wear time in older adults. *Int. J. Behav. Nutr. Phys. Act* 10:120. 10.1186/1479-5868-10-120 24156309 PMC4015851

[B37] Johansson E. Ekelund U. Nero H. Marcus C. Hagstromer M. (2015). Calibration and cross-validation of a wrist-worn actigraph in young preschoolers. *Pediatr. Obes.* 10 1–6. 10.1111/j.2047-6310.2013.00213.x 24408275

[B38] Katzmarzyk P. T. (2010). Physical activity, sedentary behavior, and health: paradigm paralysis or paradigm shift? *Diabetes* 59 2717–2725. 10.2337/db10-0822 20980470 PMC2963526

[B39] Koo T. K. Li M. Y. (2016). A guideline of selecting and reporting intraclass correlation coefficients for reliability research. *J. Chiropr. Med.* 15 155–163. 10.1016/j.jcm.2016.02.012 27330520 PMC4913118

[B40] Kramer S. Johnson L. Bernhardt J. Cumming T. (2016). Energy expenditure and cost during walking after stroke: a systematic review. *Arch. Phys. Med. Rehabil.* 97 619–632. 10.1016/j.apmr.2015.11.007 26686877

[B41] Landry G. J. Falck R. S. Beets M. W. Liu-Ambrose T. (2015). Measuring physical activity in older adults: calibrating cut-points for the MotionWatch 8© . *Front. Aging Neurosci.* 7:165. 10.3389/fnagi.2015.00165 26379546 PMC4548198

[B42] Lee J. Y. Kwon S. Kim W. S. Hahn S. J. Park J. Paik N. J. (2018). Feasibility, reliability, and validity of using accelerometers to measure physical activities of patients with stroke during inpatient rehabilitation. *PloS One* 13:e0209607. 10.1371/journal.pone.0209607 30596694 PMC6312264

[B43] Leys D. Hénon H. Mackowiak-Cordoliani M.-A. Pasquier F. (2005). Poststroke dementia. *Lancet Neurol.* 4 752–759. 10.1016/s1474-4422(05)70221-0 16239182

[B44] McDonnell M. N. (2010). Physical activity following stroke. *Arch. Phys. Med. Rehabil.* 91 665–666. 10.1016/j.apmr.2009.12.007 20382307

[B45] Moore S. A. Hallsworth K. Plötz T. Ford G. A. Rochester L. Trenell M. I. (2013). Physical activity, sedentary behaviour and metabolic control following stroke: a cross-sectional and longitudinal study. *PLoS One* 8:e55263. 10.1371/journal.pone.0055263 23383131 PMC3558428

[B46] National Institutes of Health. (2008). *Physical Activity Guidelines for Americans.* Rockville, MD: Bethseda.

[B47] Nelson M. E. Rejeski W. J. Blair S. N. Duncan P. W. Judge J. O. King A. C. (2007). Physical activity and public health in older adults: recommendation from the American college of sports medicine and the american heart association. *Med. Sci. Sports Exerc.* 49 1435–1445. 10.1249/mss.0b013e3180616aa2 17762378

[B48] Nichols J. F. Morgan C. G. Chabot L. E. Sallis J. F. Calfas K. J. (2000). Assessment of physical activity with the computer science and applications, inc., accelerometer: laboratory versus field validation. *Res. Q. Exerc. Sport* 71 36–43. 10.1080/02701367.2000.10608878 10763519

[B49] Noorkõiv M. Rodgers H. Price C. I. (2014). Accelerometer measurement of upper extremity movement after stroke: a systematic review of clinical studies. *J. Neuroeng. Rehabil.* 11:144. 10.1186/1743-0003-11-144 25297823 PMC4197318

[B50] Owen N. Healy G. N. Matthews C. E. Dunstan D. W. (2010). Too much sitting: the population-health science of sedentary behavior. *Exerc. Sports Sci. Rev.* 38 105–113. 10.1097/JES.0b013e3181e373a2 20577058 PMC3404815

[B51] Pate R. R. O’Neill J. R. Lobelo F. (2008). The evolving definition of “sedentary”. *Exerc. Sports Sci. Rev.* 36 173–178. 10.1097/JES.0b013e3181877d1a 18815485

[B52] Preiser J.-C. van Zanten A. R. Berger M. M. Biolo G. Casaer M. P. Doig G. S. (2015). Metabolic and nutritional support of critically ill patients: consensus and controversies. *Crit. Care* 19:35. 10.1186/s13054-015-0737-8 25886997 PMC4310041

[B53] Savva G. M. Stephan B. C. (2010). Epidemiological studies of the effect of stroke on incident dementia. *Stroke* 41 e41–e46. 10.1161/STROKEAHA.109.559880 19910553

[B54] Schmitz K. H. Treuth M. Hannan P. McMurray R. Ring K. B. Catellier D. (2005). Predicting energy expenditure from accelerometry counts in adolescent girls. *Med. Sci. Sports Exerc.* 37 155–161. 10.1249/01.mss.0000150084.97823.f7 15632682 PMC2491725

[B55] Schofield W. N. (1985). Predicting basal metabolic rate, new standards and review of previous work. *Hum. Nutr. Clin. Nutr.* 39 (Suppl. 1), 5–41. 4044297

[B56] Schuiling W. J. Rinkel G. J. Walchenbach R. de Weerd A. W. (2005). Disorders of sleep and wake in patients after subarachnoid hemorrhage. *Stroke* 36 578–582. 10.1161/01.str.0000154862.33213.73 15677579

[B57] Smajlovic D. Kojic B. Sinanovic O. (2006). Five-year survival-after first-ever stroke. *Bosn. J. Basic Med. Sci.* 6 17–22. 10.17305/bjbms.2006.3138 16995842 PMC7193668

[B58] Troiano R. P. McClain J. J. Brychta R. J. Chen K. Y. (2014). Evolution of accelerometer methods for physical activity research. *Br. J. Sports Med.* 48 1019–1023. 10.1136/bjsports-2014-093546 24782483 PMC4141534

