# Supplementary material for: Revisiting the MotionWatch8©: Calibrating Cut-Points for Measuring Physical Activity and Sedentary Behavior Among Adults With Stroke
Source: Front Aging Neurosci. 2019 Aug 5;11:203. doi: 10.3389/fnagi.2019.00203 (PMC6690268; doi:10.3389/fnagi.2019.00203)
Supplement: Supplementary file 1 [file Data_Sheet_1.PDF]

Supplemental Material S1. R Code

```r
#~~~~~~~~~~~~~~~~~~~~~~~~~~~~~~~~~~#
#####INITIAL DATA PROCESSING#####
#~~~~~~~~~~~~~~~~~~~~~~~~~~~~~~~~~~#

##packages##
library(psych)
library(Hmisc)
library(pastecs)
library(pROC)
library(readxl)
library(BlandAltmanLeh)
library(plyr)

#All participants included (VIT_034 Affected side only, VIT_036 Nonaffected Side)#
Nonaffected <- read_excel("Nonaffected.xlsx")
Affected <- read_excel("Affected.xlsx")

#Only participants with data on both affected and non-affected side#
Nonaffected.v2 <- read_excel("Nonaffected.v2.xlsx")
Affected.v2 <- read_excel("Affected.v2.xlsx")

##remove spaces##
spaceless <- function(x) {colnames(x) <- gsub(" ", "_", colnames(x));x}
Nonaffected <- spaceless(Nonaffected)
Affected <- spaceless(Affected)
Nonaffected.v2 <- spaceless(Nonaffected.v2)
Affected.v2 <- spaceless(Affected.v2)

##Remove ID's without Ataxia data##
Nonaffected.v2<-subset(Nonaffected.v2, Nonaffected.v2$ID!="VIT_003" &
Nonaffected.v2$ID!="VIT_027")
Affected.v2<-subset(Affected.v2, Affected.v2$ID!="VIT_003" & Affected.v2$ID!="VIT_027")

#Create BlandAltman and Reliability Data Set#
Bland.Altman1<-Nonaffected.v2[c(1,2,4,9)]
Bland.Altman2<-Affected.v2[c(1,2,4,9)]
Bland.Altman3<-merge(Bland.Altman1, Bland.Altman2, by="Output_ID")
Bland.Altman4<-Bland.Altman3[c(1,2,3,6,7)]
Bland.Altman<-rename(Bland.Altman4,c("ID.x"="ID", "Counts"="Nonaffected",
                    "Activity_Counts"="Affected", "Fugel_Meyer_Total"="FM_Total"))
Bland.Altman$Average_counts<- (Bland.Altman$Affected + Bland.Altman$Nonaffected)/2
Bland.Altman$Difference <- Bland.Altman$Affected - Bland.Altman$Nonaffected
```

```
#~~~~~~~~~~~~~~~~~~~~~~~~~~~~~~~~~~~~~~~~~~~~~~~~~~~~~~~~~#
#######BLAND ALTMAN PLOTS AND RELIABILITY ANALYSES#########
#~~~~~~~~~~~~~~~~~~~~~~~~~~~~~~~~~~~~~~~~~~~~~~~~~~~~~~~~~#

#Subset by ataxia#
Nonataxic.BlandAltman<- Bland.Altman[ which(Bland.Altman$FM_Total>= 79),]
Ataxic.BlandAltman<- Bland.Altman[ which(Bland.Altman$FM_Total< 79),]

mean(Ataxic.BlandAltman$Difference)
sd(Ataxic.BlandAltman$Difference)

#################################
#All Participants
################################

#Bland Altman#
bland.altman.plot(Bland.Altman$Affected, Bland.Altman$Nonaffected, main="All Participants (N=43)", xlab="Average Counts", ylab="Affected Counts - Unaffected Counts")

mean(Bland.Altman$Difference) #mean difference
sd(Bland.Altman$Difference)

mean(Bland.Altman$Difference) + 2*sd(Bland.Altman$Difference) #Upper Limit of Agreement
mean(Bland.Altman$Difference) - 2*sd(Bland.Altman$Difference) #Lower Limit of Agreement

#ICCAffected
ICC.All <- subset(Bland.Altman, select= c(Affected, Nonaffected))

ICC(ICC.All, missing=FALSE, alpha=0.05)

#Correlation controlling for individual
partial<-lm(Affected~Nonaffected + ID, data= Bland.Altman)
full <- lm(Affected~ID, data= Bland.Altman)
summary(partial)
summary(full)
sqrt(.5289-.2488)
#r= 0.53

#Descriptives
summary(Bland.Altman$Difference)
summary(Bland.Altman$Nonaffected)
summary(Bland.Altman$Affected)
```

```
##############################
#Mild Stroke
#############################

#Bland Altman#
bland.altman.plot(Nonataxic.BlandAltman$Affected, Nonataxic.BlandAltman$Nonaffected,
main="Adults with Mild Stroke (N= 29)", xlab="Average Counts", ylab="Affected Counts -
Unaffected Counts")

mean(Nonataxic.BlandAltman$Difference) #mean difference
sd(Nonataxic.BlandAltman$Difference)

mean(Nonataxic.BlandAltman$Difference) + 2*sd(Nonataxic.BlandAltman$Difference) #Upper
Limit of Agreement
mean(Nonataxic.BlandAltman$Difference) - 2*sd(Nonataxic.BlandAltman$Difference) #Lower
Limit of Agreement

summary(Nonataxic.BlandAltman$Difference)

#ICC
ICC.Nonataxic <- subset(Nonataxic.BlandAltman, select= c(Affected, Nonaffected))


ICC(ICC.Nonataxic, missing=FALSE, alpha=0.05)

#Correlation
partial<-lm(Affected~Nonaffected + ID, data= Nonataxic.BlandAltman)
full <- lm(Affected~ID, data= Nonataxic.BlandAltman)

summary(partial)
summary(full)
sqrt(.5045 - .2454)
#r= 0.51

#Descriptives
summary(Nonataxic.BlandAltman$Difference)
summary(Nonataxic.BlandAltman$Nonaffected)
summary(Nonataxic.BlandAltman$Affected)
```

```
##########################
#Moderate-to-Severe Stroke
##########################

#Bland Altman
bland.altman.plot(Ataxic.BlandAltman$Affected, Ataxic.BlandAltman$Nonaffected, main="Adults with
Moderate-to-Severe Stroke (N= 14)", xlab="Average Counts", ylab="Affected Counts - Unaffected Counts")

mean(Ataxic.BlandAltman$Difference) #mean difference
sd(Ataxic.BlandAltman$Difference)

mean(Ataxic.BlandAltman$Difference) + 2*sd(Ataxic.BlandAltman$Difference) #Upper Limit of Agreement
mean(Ataxic.BlandAltman$Difference) - 2*sd(Ataxic.BlandAltman$Difference) #Lower Limit of Agreement

summary(Ataxic.BlandAltman$Difference)

#ICC
ICC.Ataxic <- subset(Ataxic.BlandAltman, select= c(Affected, Nonaffected))

ICC(ICC.Ataxic, missing=FALSE, alpha=0.05)

#Correlation
partial<-lm(Affected~Nonaffected + ID, data= Ataxic.BlandAltman)
full <- lm(Affected~ID, data= Ataxic.BlandAltman)

summary(partial)
summary(full)
sqrt(.5888 - .2239)
#r= 0.60

#Descriptives
summary(Ataxic.BlandAltman$Difference)
summary(Ataxic.BlandAltman$Nonaffected)
summary(Ataxic.BlandAltman$Affected)
```

#~~~~~~#
##ALL PARTICIPANT DATA INCLUDED IN ROC CURVES##
#~~~~~~#

#########Sedentary Behaviour###########
rocSB.nonaffected <- roc(Nonaffected.v2$SB, Nonaffected.v2$Counts, percent=TRUE)
#Nonaffected
rocSB.affected <- roc(Affected.v2$SB, Affected.v2$Activity_Counts, percent=TRUE)
#Affected

plot(rocSB.nonaffected, col = 1, lty = 1, main = "Sedentary Behaviour")
plot(rocSB.affected, col = 1, lty = 2, add= TRUE)

##Confidence Interval for AUC##
roc(Nonaffected.v2$SB, Nonaffected.v2$Counts, ci=TRUE, of="auc", boot.n=200, ci.alpha= 0.95,
stratified=FALSE) #Nonaffected
roc(Affected.v2$SB, Affected.v2$Activity_Counts, ci=TRUE, of="auc", boot.n=200, ci.alpha= 0.95,
stratified=FALSE) #Affected

##Best Fit Youden index##

#Nonaffected#
coords(rocSB.nonaffected, "b", ret = "t", best.method= "youden")
coords(rocSB.nonaffected, 164.5, ret=c("accuracy","specificity", "sensitivity", "threshold", "ppv", "npv"))
69.18+(75.15-100)#Youden Index

#Affected#
coords(rocSB.affected, "b", ret = "t", best.method= "youden")
coords(rocSB.affected, 133.5, ret=c("accuracy","specificity", "sensitivity", "threshold", "ppv", "npv", "youden"))
78.77+(70.58-100)#Youden Index

#############Moderate-to-Vigorous Physical Activity###############
rocMVPA.nonaffected <- roc(Nonaffected.v2$MVPA, Nonaffected.v2$Counts, percent=TRUE)
#Nonaffected
rocMVPA.affected <- roc(Affected.v2$MVPA, Affected.v2$Activity_Counts, percent=TRUE)
#Affected

plot(rocMVPA.nonaffected, col = 1, lty = 1, main = "Moderate-to-Vigorous Physical Activity")
plot(rocMVPA.affected, col = 1, lty = 2, add= TRUE)

##Confidence Interval for AUC##

```
roc(Nonaffected.v2$MVPA, Nonaffected.v2$Counts, ci=TRUE, of="auc", boot.n=200,
ci.alpha= 0.95,
    stratified=FALSE) #Nonaffected
roc(Affected.v2$MVPA, Affected.v2$Activity_Counts, ci=TRUE, of="auc", boot.n=200,
ci.alpha= 0.95,
    stratified=FALSE) #Affected
```

######ROC Curve for False Positive Ratio Less than 0.1####

#Nonaffected

```
specificity.nonaffected <- coords(rocMVPA.nonaffected, "local maximas", "specificity",
as.matrix=TRUE)
MVPA_Subset.nonaffected <- as.matrix(print(specificity.nonaffected))
MVPA_sub.nonaffected <- as.data.frame(t(specificity.nonaffected))
MVPA_Subset.nonaffected <- MVPA_sub.nonaffected[
which(MVPA_sub.nonaffected$specificity > 90),]
MVPA_Subset.nonaffected$Youden <- MVPA_Subset.nonaffected$sensitivity +
MVPA_Subset.nonaffected$specificity - 100
View(MVPA_Subset.nonaffected)

coords(rocMVPA.affected, 685, ret=c("accuracy","specificity", "sensitivity", "threshold", "ppv",
"npv", "youden"))
```

#Affected

```
specificity.affected <- coords(rocMVPA.affected, "local maximas", "specificity",
as.matrix=TRUE)
MVPA_Subset.affected <- as.matrix(print(specificity.affected))
MVPA_sub.affected <- as.data.frame(t(specificity.affected))
MVPA_Subset.affected <- MVPA_sub.affected[ which(MVPA_sub.affected$specificity > 90),]
MVPA_Subset.affected$Youden <- MVPA_Subset.affected$sensitivity +
MVPA_Subset.affected$specificity - 100
View(MVPA_Subset.affected)

coords(rocMVPA.affected, 650, ret=c("accuracy","specificity", "sensitivity", "threshold", "ppv",
"npv", "youden"))
```

#Significantly different roc curves for all participants#

```
roc.test(rocSB.nonaffected, rocSB.affected) #Significant (p<0.01)
roc.test(rocMVPA.nonaffected, rocMVPA.affected) #Significant (p<0.01)
```

```
#~~~~~~~~~~~~~~~~~~~~~~~~~~~~~~~~~~~~~~~~~~~~~~~~~~~~~~~~~~~~~~#
###############SUBSET ROC-CURVES FOR BASED ON ATAXIA#################
#~~~~~~~~~~~~~~~~~~~~~~~~~~~~~~~~~~~~~~~~~~~~~~~~~~~~~~~~~~~~~~#
Nonaffected_Healthy <- Nonaffected.v2[ which(Nonaffected.v2$FM_Total >=79),]
Nonaffected_Ataxic <- Nonaffected.v2[ which(Nonaffected.v2$FM_Total <79),]
Affected_Healthy <- Affected.v2[ which(Affected.v2$Fugel_Meyer_Total >= 79),]
Affected_Ataxic <- Affected.v2[ which(Affected.v2$Fugel_Meyer_Total < 79),]

############NON-ATAXIC#################
#~~~~~~~~~~~~~~~~~~~~~~~~~~~~~~~~~~~#

####SEDENTARY BEHAVIOUR####
rocSB.nonaffected.nonataxic <- roc(Nonaffected_Healthy$SB, Nonaffected_Healthy$Counts,
percent=TRUE) #Nonaffected
rocSB.affected.nonataxic <- roc(Affected_Healthy$SB, Affected_Healthy$Activity_Counts,
percent=TRUE) #Affected

plot(rocSB.nonaffected.nonataxic, col = 1, lty = 1, main = "Sedentary Behaviour")
plot(rocSB.affected.nonataxic, col = 1, lty = 2, add= TRUE)

##Confidence Interval for AUC##
roc(Nonaffected_Healthy$SB, Nonaffected_Healthy$Counts, ci=TRUE, of="auc", boot.n=200,
ci.alpha= 0.95,
   stratified=FALSE) #Nonaffected
roc(Affected_Healthy$SB, Affected_Healthy$Activity_Counts, ci=TRUE, of="auc",
boot.n=200, ci.alpha= 0.95,
   stratified=FALSE) #Affected

##Best Fit Youden index##
#Nonaffected#
coords(rocSB.nonaffected.nonataxic, "b", ret = "t", best.method= "youden")
coords(rocSB.nonaffected.nonataxic, 162, ret=c("accuracy","specificity", "sensitivity",
"threshold", "ppv", "npv", "youden"))
73.26+76.27-100 #Youden Index

#Affected#
coords(rocSB.affected.nonataxic, "b", ret = "t", best.method= "youden")
coords(rocSB.affected.nonataxic, 133.5, ret=c("accuracy","specificity", "sensitivity",
"threshold", "ppv", "npv", "youden"))
74.98+75.35-100 #Youden Index
```

#########Moderate-to-Vigorous Physical Activity###############

```
rocMVPA.nonaffected.nonataxic <- roc(Nonaffected_Healthy$MVPA,
Nonaffected_Healthy$Counts, percent=TRUE) #Nonaffected
rocMVPA.affected.nonataxic <- roc(Affected_Healthy$MVPA,
Affected_Healthy$Activity_Counts, percent=TRUE) #Affected

plot(rocMVPA.nonaffected, col = 1, lty = 1, main = "Moderate-to-Vigorous Physical Activity")
plot(rocMVPA.affected, col = 1, lty = 2, add= TRUE)
```

##Confidence Interval for AUC##

```
roc(Nonaffected_Healthy$MVPA, Nonaffected_Healthy$Counts, ci=TRUE, of="auc",
boot.n=200, ci.alpha= 0.95,
    stratified=FALSE) #Nonaffected
roc(Affected_Healthy$MVPA, Affected_Healthy$Activity_Counts, ci=TRUE, of="auc",
boot.n=200, ci.alpha= 0.95,
    stratified=FALSE) #Affected
```

###ROC Curve for False Positive Ratio Less than 0.1###

#Nonaffected

```
specificity.nonaffected.healthy <- coords(rocMVPA.nonaffected.nonataxic, "local maximas",
"specificity", as.matrix=TRUE)
MVPA_Subset.nonaffected.healthy <- as.matrix(print(specificity.nonaffected.healthy))
MVPA_sub.nonaffected.healthy <- as.data.frame(t(specificity.nonaffected.healthy))
MVPA_Subset.nonaffected.healthy <- MVPA_sub.nonaffected.healthy[
which(MVPA_sub.nonaffected.healthy$specificity > 90),]
MVPA_Subset.nonaffected.healthy$Youden <- MVPA_Subset.nonaffected.healthy$sensitivity +
MVPA_Subset.nonaffected.healthy$specificity - 100
View(MVPA_Subset.nonaffected.healthy)

coords(rocMVPA.nonaffected.nonataxic, 660.5, ret=c("accuracy","specificity", "sensitivity",
"threshold", "ppv", "npv", "youden"))
```

#Affected

```
specificity.affected.healthy <- coords(rocMVPA.affected.nonataxic, "local maximas",
"specificity", as.matrix=TRUE)
MVPA_Subset.affected.healthy <- as.matrix(print(specificity.affected.healthy))
MVPA_sub.affected.healthy <- as.data.frame(t(specificity.affected.healthy))
MVPA_Subset.affected.healthy <- MVPA_sub.affected.healthy[
which(MVPA_sub.affected.healthy$specificity > 90),]
MVPA_Subset.affected.healthy$Youden <- MVPA_Subset.affected.healthy$sensitivity +
MVPA_Subset.affected.healthy$specificity - 100
```

View(MVPA_Subset.affected.healthy)

coords(rocMVPA.affected.nonataxic, 704, ret=c("accuracy","specificity", "sensitivity", "threshold", "ppv", "npv", "youden"))

#Significantly different roc curves for nonataxic participants#
roc.test(rocSB.nonaffected.nonataxic, rocSB.affected.nonataxic)
roc.test(rocMVPA.nonaffected.nonataxic, rocMVPA.affected.nonataxic)

```
#~~~~~~~~~~~~~~~~~~~~~~~~~~~~~~~~#
###########ATAXIC################
#~~~~~~~~~~~~~~~~~~~~~~~~~~~~~~~~#

####SEDENTARY BEHAVIOUR####
rocSB.nonaffected.ataxic <- roc(Nonaffected_Ataxic$SB, Nonaffected_Ataxic$Counts,
percent=TRUE) #Nonaffected
rocSB.affected.ataxic <- roc(Affected_Ataxic$SB, Affected_Ataxic$Activity_Counts,
percent=TRUE) #Affected

plot(rocSB.nonaffected.ataxic, col = 1, lty = 1, main = "Sedentary Behaviour")
plot(rocSB.affected.ataxic, col = 1, lty = 2, add= TRUE)

##Confidence Interval for AUC##
roc(Nonaffected_Ataxic$SB, Nonaffected_Ataxic$Counts, ci=TRUE, of="auc", boot.n=200,
ci.alpha= 0.95,
    stratified=FALSE) #Nonaffected
roc(Affected_Ataxic$SB, Affected_Ataxic$Activity_Counts, ci=TRUE, of="auc", boot.n=200,
ci.alpha= 0.95,
    stratified=FALSE) #Affected

##Best Fit Youden index##

#Nonaffected#
coords(rocSB.nonaffected.ataxic, "b", ret = "t", best.method= "youden")
coords(rocSB.nonaffected.ataxic, 280.5, ret=c("accuracy","specificity", "sensitivity",
"threshold", "ppv", "npv", "youden"))
78+61-100 #Youden Index

#Affected#
coords(rocSB.affected.ataxic, "b", ret = "t", best.method= "youden")
coords(rocSB.affected.ataxic, 123, ret=c("accuracy","specificity", "sensitivity", "threshold",
"ppv", "npv", "youden"))
63+85.33-100 #Youden Index
```

```
########Moderate-to-Vigorous Physical Activity##############
rocMVPA.nonaffected.ataxic <- roc(Nonaffected_Ataxic$MVPA, Nonaffected_Ataxic$Counts,
percent=TRUE) #Nonaffected
rocMVPA.affected.ataxic <- roc(Affected_Ataxic$MVPA, Affected_Ataxic$Activity_Counts,
percent=TRUE) #Affected

plot(rocMVPA.nonaffected.ataxic, col = 1, lty = 1, main = "Moderate-to-Vigorous Physical
Activity")
plot(rocMVPA.affected.ataxic, col = 1, lty = 2, add= TRUE)

##Confidence Interval for AUC##
roc(Nonaffected_Ataxic$MVPA, Nonaffected_Ataxic$Counts, ci=TRUE, of="auc", boot.n=200,
ci.alpha= 0.95,
   stratified=FALSE) #Nonaffected
roc(Affected_Ataxic$MVPA, Affected_Ataxic$Activity_Counts, ci=TRUE, of="auc",
boot.n=200, ci.alpha= 0.95,
   stratified=FALSE) #Affected

###ROC Curve for False Positive Ratio Less than 0.1###

#Nonaffected
specificity.nonaffected.ataxic <- coords(rocMVPA.nonaffected.ataxic, "local maximas",
"specificity", as.matrix=TRUE)
MVPA_Subset.nonaffected.ataxic <- as.matrix(print(specificity.nonaffected.ataxic))
MVPA_sub.nonaffected.ataxic <- as.data.frame(t(specificity.nonaffected.ataxic))
MVPA_Subset.nonaffected.ataxic <- MVPA_sub.nonaffected.ataxic[
which(MVPA_sub.nonaffected.ataxic$specificity > 90),]
MVPA_Subset.nonaffected.ataxic$Youden <- MVPA_Subset.nonaffected.ataxic$sensitivity +
MVPA_Subset.nonaffected.ataxic$specificity - 100
View(MVPA_Subset.nonaffected.ataxic)

coords(rocMVPA.nonaffected.ataxic, 737.5, ret=c("accuracy","specificity", "sensitivity",
"threshold", "ppv", "npv", "youden"))

#Affected
specificity.affected.ataxic <- coords(rocMVPA.affected.ataxic, "local maximas", "specificity",
as.matrix=TRUE)
MVPA_Subset.affected.ataxic <- as.matrix(print(specificity.affected.ataxic))
MVPA_sub.affected.ataxic <- as.data.frame(t(specificity.affected.ataxic))
MVPA_Subset.affected.ataxic <- MVPA_sub.affected.ataxic[
which(MVPA_sub.affected.ataxic$specificity > 90),]
MVPA_Subset.affected.ataxic$Youden <- MVPA_Subset.affected.ataxic$sensitivity +
MVPA_Subset.affected.ataxic$specificity - 100
```

View(MVPA_Subset.affected.ataxic)

coords(rocMVPA.affected.ataxic, 468, ret=c("accuracy","specificity", "sensitivity", "threshold", "ppv", "npv", "youden"))

#Significantly different roc curves for ataxic participants#
roc.test(rocSB.nonaffected.ataxic, rocSB.affected.ataxic)
roc.test(rocMVPA.nonaffected.ataxic, rocMVPA.affected.ataxic)
